# Rhino-Orbital Mucormycosis Following COVID-19 Viral Vector Vaccination in an Immunocompetent Patient

**DOI:** 10.3390/jof12070516

**Published:** 2026-07-14

**Authors:** Diego Strianese, Mario Troisi, Adriana Iuliano, Dana Cohen, Francesco Matarazzo, Maria Paola Laezza, Biagio Pinchera, Maria Laura Passaro, Davide Tramontano, Vittoria Lanni, Antonella D’Aponte, Ivan Gentile, Ciro Costagliola

**Affiliations:** 1 Ophthalmology Unit, Department of Neurosciences, Reproductive Sciences and Dentistry, University of Naples Federico II, 80131 Naples, Italy; diego.strianese@unina.it (D.S.); adrianaiuliano@yahoo.it (A.I.); farncesco.matarazzo@unina.it (F.M.); m.laura.passaro@gmail.com (M.L.P.); davidetramontano40@gmail.com (D.T.); vittorialanni@hotmail.com (V.L.); antodaponte@yahoo.it (A.D.); ciro.costagliola@unina.it (C.C.); 2 Ophthalmology Unit, Galilee Medical Center, Nahariya 22100, Israel; cohen.dana19@gmail.com; 3 Department of Physics “Ettore Pancini”, University of Naples Federico II, 80126 Naples, Italy; 4 Ophthalmology Unit, Ospedale del Mare, ASL Napoli 1-centro, 80147 Naples, Italy; mariap.laezza@gmail.com; 5 Infectious Diseases Unit, Department of Clinical Medicine and Surgery, University of Naples Federico II, 80131 Naples, Italy; biagio.pinchera@unina.it (B.P.); ivan.gentile@unina.it (I.G.); 6 Department of Medicine and Health Sciences “V. Tiberio”, University of Molise, 86100 Campobasso, Italy

**Keywords:** rhino-orbital mucormycosis, COVID-19 vaccine, AZD1222, orbital cellulitis, opportunistic fungal infection, immunocompetent host

## Abstract

Rhino-orbital mucormycosis is a rare, life-threatening opportunistic fungal infection, typically affecting immunocompromised patients. During the COVID-19 pandemic, increased cases were mainly linked to SARS-CoV-2 infection, diabetes, and corticosteroid exposure. We report a severe case in a previously healthy 44-year-old immunocompetent man who developed acute left-sided exophthalmos, ophthalmoplegia, severe visual loss, and systemic deterioration 10 days after AZD1222 COVID-19 vaccination. Clinical and radiologic findings suggested invasive rhino-orbital fungal disease, prompting immediate liposomal amphotericin B, broad-spectrum antibiotics, urgent endoscopic sinus surgery, and repeated orbital–sinonasal debridements with amphotericin B irrigation. Histopathological examination demonstrated broad aseptate hyphae with tissue necrosis, consistent with mucormycosis, while fungal culture and ITS sequencing identified *Rhizopus arrhizus* as the causative species. Therapy was later adjusted to include isavuconazole and antibacterial coverage for persistent inflammation and secondary colonization. Orbital and systemic improvement occurred within the first week, with globe preservation and marked proptosis reduction at 6 months, despite persistent ophthalmoplegia and residual light perception. Isavuconazole was continued for 2 years, with no recurrence during 3 years of follow-up. Although causality with vaccination cannot be established, the temporal association and biological plausibility warrant further investigation. Early suspicion and prompt combined medical–surgical management are essential in rapidly progressive orbital cellulitis.

## 1. Introduction

Mucormycosis is a rare but life-threatening invasive fungal infection caused by filamentous fungi of the order Mucorales, typically affecting immunocompromised individuals [1]. Major predisposing risk factors include diabetes mellitus (DM), particularly in the setting of poor metabolic control, hematologic or solid malignancies, solid organ or hematopoietic stem cell transplantation, severe neutropenia, primary or acquired immunodeficiencies, and prolonged corticosteroid or immunosuppressive therapy [2]. Among its clinical forms, rhino-orbital mucormycosis is one of the most aggressive presentations, involving the nasal cavity, paranasal sinuses, orbit, and potentially the brain through rapid angioinvasive spread [1]. The most common presentation is rhino-orbito-cerebral mucormycosis (ROCM), characterized by orbital pain, periorbital swelling, facial pain or numbness, conjunctival chemosis, visual impairment, progressive ophthalmoplegia, blindness, and, in severe cases, cavernous sinus thrombosis and intracranial extension [1,3].

Since the COVID-19 pandemic, a marked increase in mucormycosis cases has been reported worldwide, particularly among patients with active or previous SARS-CoV-2 infection [4]. Several mechanisms have been proposed to explain this association, including virus-induced immune dysregulation, endothelial damage, hyperferritinemia, uncontrolled hyperglycemia, and corticosteroid exposure [4]. In parallel, SARS-CoV-2 infection has been associated with multiple ocular manifestations, including conjunctivitis, episcleritis, uveitis, retinal vascular abnormalities, optic neuritis, cranial nerve palsies, ocular myasthenia gravis, and acute dacryoadenitis [5,6,7,8].

Furthermore, ocular inflammatory adverse events have also been reported following COVID-19 vaccination, including conjunctival and eyelid reactions, optic neuritis, and intraocular inflammation [9]. The overlap between post-vaccination ocular findings and ocular manifestations observed during COVID-19 infection has led to the hypothesis of shared immune-mediated mechanisms, potentially involving Toll-like receptor (TLR) activation and innate immune dysregulation [10]. We report a rare case of rhino-orbital mucormycosis in an apparently immunocompetent patient, in whom symptoms developed 10 days after administration of the AZD1222 (Oxford–AstraZeneca) viral vector COVID-19 vaccine.

## 2. Case Report

A 44-year-old previously healthy Caucasian man presented in April 2021 to a local Emergency Department in Southern Italy with progressive left hemifacial swelling associated with erythema, pain, tenderness, and local warmth. Initial computed tomography (CT) imaging demonstrated inflammatory infiltration of the left orbital fat and bilateral maxillary sinus opacification. The patient denied recent trauma and had no history of diabetes mellitus, hematologic malignancy, chronic corticosteroid therapy, organ transplantation, or other significant systemic comorbidities. Laboratory investigations at presentation demonstrated a normal platelet count (268 × 10^9^/L; reference range 150–450 × 10^9^/L) and normal coagulation parameters, including prothrombin time (PT) of 12.1 s (reference range 11.0–13.5 s), international normalized ratio (INR) of 1.02 (reference range 0.8–1.2), activated partial thromboplastin time (aPTT) of 30.4 s (reference range 25–35 s), fibrinogen of 342 mg/dL (reference range 200–400 mg/dL), and D-dimer of 0.38 mg/L FEU (reference range <0.50 mg/L FEU), with no evidence of thrombocytopenia or coagulopathy. Fasting blood glucose was 92 mg/dL (reference range 70–99 mg/dL), glycated hemoglobin (HbA1c) was 5.3% (reference range <5.7%), and the absolute neutrophil count was 4.8 × 10^9^/L (reference range 1.5–7.5 × 10^9^/L). HIV serology was negative. No clinical or laboratory findings suggestive of an underlying immunodeficiency were identified. Family history was unremarkable. Medical history was notable for administration of a single dose of the AZD1222 (Oxford–AstraZeneca) COVID-19 vaccine 10 days before symptom onset. One week before admission, the patient had undergone dental extraction for a contralateral root perforation. A nasopharyngeal SARS-CoV-2 polymerase chain reaction (PCR) test performed at admission was negative.

Two days after admission, the patient experienced sudden severe visual deterioration in the left eye and underwent urgent functional endoscopic sinus surgery (FESS) with left maxillary sinus drainage, removal of inflammatory polyps, and sinus decompression. Empirical broad-spectrum antibiotic therapy with piperacillin/tazobactam (4.5 g three times daily) and linezolid (600 mg twice daily) was initiated. Despite 5 days of treatment, clinical deterioration progressed, prompting transfer to a tertiary referral center for orbital disease management.

**Figure 1:**
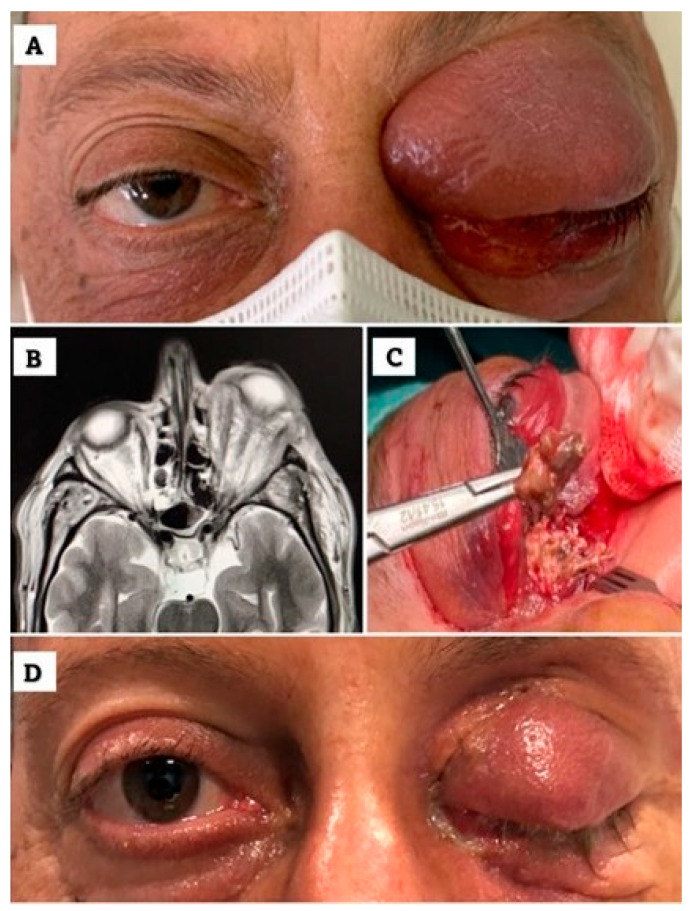
Clinical presentation, orbital imaging, surgical findings, and post-treatment outcome in an immunocompetent patient with rhino-orbital mucormycosis following AZD1222 (Oxford–AstraZeneca) COVID-19 vaccination. (**A**) Clinical appearance at presentation showing severe left-sided proptosis, complete ptosis, and diffuse periorbital edema. (**B**) Orbital magnetic resonance imaging (MRI) demonstrating involvement of the left orbital apex with necrotic soft tissue changes, diffuse orbital fat infiltration, and thickening of the optic nerve sheath. (**C**) Intraoperative image obtained during orbital debridement showing extensive necrotic tissue removal and surgical exploration of the orbital cavity. (**D**) Clinical appearance at discharge showing marked reduction in proptosis and chemosis, with persistent ptosis and ophthalmoplegia.

On admission, ophthalmologic examination revealed complete ophthalmoplegia of the left eye (OS), moderate proptosis, complete ptosis with absent levator function, fixed mydriasis without pupillary reflexes, and no light perception (Figure 1A). The right eye (OD) was unremarkable. Slit-lamp examination showed severe conjunctival chemosis and exposure-related corneal epitheliopathy in the left eye. Fundus examination demonstrated optic disc edema and diffuse retinal pallor. Neurological examination revealed ipsilateral facial hypoesthesia, confusion, impaired coordination, and fluctuating consciousness.

Orbital magnetic resonance imaging (MRI) demonstrated enlargement of the left orbital fissure with extensive inflammatory infiltration of the orbital apex, necrotic soft tissue changes, diffuse orbital fat involvement, thickening of the left optic nerve sheath, and enhancement of the ipsilateral inferior rectus muscle (Figure 1B). Based on the rapidly progressive clinical course and radiologic findings, invasive rhino-orbital mucormycosis was strongly suspected after multidisciplinary evaluation.

Empirical treatment with high-dose meropenem (2 g every 8 h), linezolid (600 mg every 12 h), and liposomal amphotericin B (5 mg/kg/day) was initiated immediately, before histopathologic confirmation. Laboratory markers subsequently demonstrated gradual normalization of leukocyte count and inflammatory indices. Three days later, the patient underwent an orbitotomy with extensive drainage and debridement of necrotic orbital tissue combined with additional sinus surgery. A lateral canthotomy and inferior cantholysis were performed to improve orbital decompression. Necrotic tissue biopsy specimens were obtained for microbiological and histopathological analysis (Figure 1C), and intraoperative amphotericin B lavage (1 mg/mL) was administered.

Histopathology demonstrated broad, ribbon-like aseptate fungal hyphae with right-angle branching associated with extensive tissue necrosis and granulomatous inflammation, confirming mucormycosis. Microbiological investigations excluded alternative fungal pathogens, including Aspergillus spp. Fungal culture of the surgical specimen yielded a Mucorales isolate. Species-level identification was established by sequencing the internal transcribed spacer (ITS) region of the ribosomal DNA. The ITS region was amplified using the universal primers ITS1 (5′-TCCGTAGGTGAACCTGCGG-3′) and ITS4 (5′-TCCTCCGCTTATTGATATGC-3′), and the resulting amplicons underwent bidirectional Sanger sequencing. Sequence analysis demonstrated >99% identity with *Rhizopus arrhizus* (formerly *Rhizopus oryzae*), confirming the etiological diagnosis of rhino-orbital mucormycosis. Follow-up MRI performed 5 days after surgery showed reduction in orbital inflammation, improvement of exophthalmos, and decreased compression of the optic nerve, although inflammatory changes persisted within the frontal sinus. Repeated sinus drainage and local irrigations with aspiration of purulent material were subsequently required.

Within the first week, marked improvement in orbital and systemic conditions was observed, including recovery of neurological responsiveness and improvement of ipsilateral hearing impairment. However, the subsequent clinical course remained prolonged and fluctuating. Four weeks later, despite three orbital debridements, the patient developed recurrent proptosis, complete ophthalmoplegia, worsening ptosis, inferior globe displacement, persistent mydriasis, and conjunctival chemosis. Antimicrobial therapy was escalated to include metronidazole (500 mg three times daily), oral isavuconazole (loading dose followed by 200 mg/day), and daptomycin (6 mg/kg/day), while linezolid was discontinued. Due to persistent fever, elevated inflammatory markers, and rectal colonization with carbapenemase-producing organisms, meropenem was replaced with ceftazidime/avibactam (2.5 g three times daily).

On day 44 of liposomal amphotericin B therapy, progressive clinical improvement became evident, with resolution of fever, reduction in inflammatory markers, and significant improvement of proptosis and chemosis. Antibiotics were discontinued, whereas antifungal treatment with liposomal amphotericin B and isavuconazole was maintained. During this phase, the patient developed amphotericin B-related adverse effects, including hypokalemia requiring oral potassium supplementation and gastrointestinal intolerance with nausea and vomiting. Subsequent MRI revealed a residual orbital collection requiring additional surgical drainage.

After 71 days of hospitalization, the patient was discharged in stable condition. Intravenous isavuconazole was transitioned to oral therapy (200 mg/day). At discharge, best-corrected visual acuity was hand motion in the left eye and 20/20 in the right eye, with marked regression of proptosis and chemosis but persistent complete ptosis and ophthalmoplegia (Figure 1D). Oral isavuconazole maintenance therapy was continued for 2 years. At 1-year follow-up after discontinuation of antifungal treatment, the patient remained clinically stable without evidence of recurrent infection.

## 3. Discussion

This case describes severe rhino-orbital mucormycosis (ROCM) occurring shortly after AZD1222 (Oxford–AstraZeneca) COVID-19 vaccination in an apparently immunocompetent patient without diabetes mellitus, hematologic malignancy, chronic corticosteroid exposure, organ transplantation, neutropenia, or active SARS-CoV-2 infection. Such a presentation is unusual, as ROCM is classically associated with impaired innate immunity, particularly uncontrolled diabetes, ketoacidosis, hematologic malignancies, prolonged corticosteroid therapy, or transplant-related immunosuppression [1,11]. However, the absence of recognized predisposing factors does not exclude the diagnosis. In a comprehensive review by Elinav et al., approximately 9% (95% CI, 6.7–11.8%) of reported rhinocerebral mucormycosis cases occurred in patients without known underlying risk factors, emphasizing that this infection may occasionally develop in apparently immunocompetent individuals and that the absence of classical risk factors should not delay clinical suspicion when compatible features are present [12]. The rapid progression of orbital and sinonasal involvement observed in this patient is consistent with the well-recognized angioinvasive nature of Mucorales infection [13].

Mucormycosis is characterized by endothelial invasion leading to vascular thrombosis, ischemia, and tissue necrosis [14]. Experimental studies have demonstrated that Mucorales express CotH proteins capable of binding endothelial GRP78 receptors, thereby facilitating angioinvasion and tissue dissemination [14,15]. Host defense relies predominantly on neutrophil and macrophage activity, with macrophages inhibiting spore germination and neutrophils mediating hyphal destruction through oxidative and non-oxidative mechanisms [14]. Consequently, disturbances in endothelial integrity or innate immune regulation may theoretically facilitate fungal invasion in susceptible tissues. In the present case, molecular analysis identified *Rhizopus arrhizus*, the predominant etiological agent of rhino-orbital and rhino-orbito-cerebral mucormycosis worldwide, accounting for approximately 50–70% of reported cases [11,14]. This species is recognized for its high virulence, rapid tissue invasion, and propensity for angioinvasive disease, making it the leading cause of invasive sino-orbital mucormycosis. The identification of *R. arrhizus* therefore strengthens the microbiological diagnosis and is consistent with the fulminant clinical course observed in our patient despite the absence of classical systemic predisposing factors.

During the COVID-19 pandemic, mucormycosis gained considerable attention because of the emergence of COVID-19-associated mucormycosis (CAM), particularly in patients with uncontrolled diabetes, corticosteroid exposure, hypoxia, and systemic inflammatory dysregulation [4,16]. In contrast, the present case occurred in temporal association with AZD1222 viral vector COVID-19 vaccination rather than active SARS-CoV-2 infection. This distinction is important because the patient lacked the major systemic predisposing factors typically observed in CAM.

AZD1222 is an adenoviral vector vaccine capable of inducing robust innate and adaptive immune activation through cytokine signaling, endothelial activation, and cellular immune responses [17]. Rare thromboinflammatory and immune-mediated adverse events associated with adenoviral vector vaccination, including vaccine-induced immune thrombotic thrombocytopenia (VITT), vascular occlusive phenomena, and inflammatory ocular complications, have been described [18]. Although these events remain exceptionally uncommon, they support the possibility that transient endothelial or inflammatory dysregulation may occur in susceptible individuals.

This observation may be relevant in the context of ROCM, where endothelial injury and microvascular thrombosis represent central pathogenic mechanisms [14]. Although the close temporal relationship between vaccination and symptom onset raises the possibility of a biological link, temporal association alone is insufficient to establish causality. The present report does not support generalized vaccine-induced immunosuppression. If an association exists, a more plausible explanation would involve transient immune-endothelial dysregulation acting together with local tissue susceptibility rather than a direct causal vaccine effect.

The preceding dental extraction may represent an additional local predisposing factor. Dental and maxillary procedures can induce mucosal disruption, local inflammation, and transient alteration of sinonasal microbial balance, potentially facilitating fungal invasion in anatomically vulnerable regions [19,20]. In the present patient, the most balanced interpretation is therefore multifactorial, with recent vaccination possibly acting as a transient systemic trigger in the presence of local tissue vulnerability after dental manipulation.

The literature describing mucormycosis after COVID-19 vaccination remains extremely limited. Shah et al. reported a case of primary cutaneous mucormycosis developing at the injection site after mRNA-1273 vaccination in a patient with bullous pemphigoid, where local tissue disruption was considered the most likely facilitating mechanism [21]. Although anatomically different from the present case, that report demonstrates that mucormycosis temporally associated with COVID-19 vaccination has previously been documented. Other published reports are generally confounded by immunosuppression, diabetes, corticosteroid exposure, or concomitant SARS-CoV-2 infection, limiting causal interpretation.

The clinical course of our patient also highlights the aggressive nature of ROCM and emphasizes the importance of considering invasive fungal disease early in the differential diagnosis of rapidly progressive orbital or sinonasal inflammation. Although definitive diagnosis was established only after referral to our tertiary care center, initiation of systemic liposomal amphotericin B, repeated surgical debridement, and close radiological monitoring resulted in infection control and globe preservation without orbital exenteration, despite irreversible visual loss. This outcome underscores the value of coordinated multidisciplinary management once the diagnosis is recognized, while also reflecting the well-established association between delayed diagnosis and poorer clinical outcomes in ROCM [3].

Overall, this case contributes to the limited literature describing mucormycosis outside conventional risk categories. The purpose is not to imply a confirmed causal relationship between COVID-19 vaccination and mucormycosis, but rather to document a rare temporal association characterized by biological plausibility and absence of traditional systemic predisposing factors. Further pharmacovigilance data and mechanistic studies would be necessary before any causal inference could be established.

## 4. Conclusions

This report describes a rare case of rhino-orbital mucormycosis occurring shortly after AZD1222 COVID-19 vaccination in an apparently immunocompetent patient without classical systemic risk factors. Although the temporal relationship is noteworthy, it does not establish causality, and the potential contribution of vaccination to disease development remains uncertain. This case expands the limited literature on rhino-orbital mucormycosis occurring outside conventional risk categories and highlights the need for continued pharmacovigilance and further epidemiological and mechanistic studies. Clinicians should maintain a high index of suspicion for invasive fungal disease in patients presenting with rapidly progressive orbital or sinonasal inflammation, even in the absence of traditional predisposing conditions. Early recognition, prompt antifungal therapy, aggressive surgical management, and multidisciplinary care remain essential to improve outcomes in ROCM.

## Data Availability

No datasets were generated or analysed during the current study.

## Abbreviations

The following abbreviations are used in this manuscript:

AZD1222	Oxford–AstraZeneca COVID-19 vaccine
BCVA	Best-corrected visual acuity
CAM	COVID-19-associated mucormycosis
COVID-19	Coronavirus disease 2019
CT	Computed tomography
DM	Diabetes mellitus
FESS	Functional endoscopic sinus surgery
GRP78	Glucose-regulated protein 78
MRI	Magnetic resonance imaging
OD	Right eye
OS	Left eye
PCR	Polymerase chain reaction
ROCM	Rhino-orbito-cerebral mucormycosis
ROCM/ROM	Rhino-orbital/rhino-orbito-cerebral mucormycosis
SARS-CoV-2	Severe acute respiratory syndrome coronavirus 2
TLR	Toll-like receptor
VITT	Vaccine-induced immune thrombotic thrombocytopenia
